# Association mapping and candidate genes for physiological non-destructive traits: Chlorophyll content, canopy temperature, and specific leaf area under normal and saline conditions in wheat

**DOI:** 10.3389/fgene.2022.980319

**Published:** 2022-09-30

**Authors:** Alaa A. Said, Yasser S. Moursi, Ahmed Sallam

**Affiliations:** ^1^ Department of Agronomy, Faculty of Agriculture, Sohag University, Egypt; ^2^ Department of Botany, Faculty of Science, Fayoum University, Fayoum, Egypt; ^3^ Resources Genetics and Reproduction, Department Genebank, Leibniz Institute of Plant Genetics and Crop Plant Research (IPK), Stadt Seeland, Germany; ^4^ Department of Genetics, Faculty of Agriculture, Assiut University, Assiut, Egypt

**Keywords:** *Triticum aestivum* L., GWAS, salinity, physiological traits, candidate genes

## Abstract

Wheat plants experience substantial physiological adaptation when exposed to salt stress. Identifying such physiological mechanisms and their genetic control is especially important to improve its salt tolerance. In this study, leaf chlorophyll content (CC), leaf canopy temperature (CT), and specific leaf area (SLA) were scored in a set of 153 (103 having the best genotypic data were used for GWAS analysis) highly diverse wheat genotypes under control and salt stress. On average, CC and SLA decreased under salt stress, while the CT average was higher under salt stress compared to the control. CT was negatively and significantly correlated with CC under both conditions, while no correlation was found between SLA and CC and CT together. High genetic variation and broad-sense-heritability estimates were found among genotypes for all traits. The genome wide association study revealed important QTLs for CC under both conditions (10) and SLA under salt stress (four). These QTLs were located on chromosomes 1B, 2B, 2D, 3A, 3B, 5A, 5B, and 7B. All QTLs detected in this study had major effects with R^2^ extending from 20.20% to 30.90%. The analysis of gene annotation revealed three important candidate genes (*TraesCS5A02G355900*, *TraesCS1B02G479100*, and *TraesCS2D02G509500*). These genes are found to be involved in the response to salt stress in wheat with high expression levels under salt stress compared to control based on mining in data bases.

## Introduction

Wheat (*Triticum aestivum* L.) is the most important strategic crop, with a production of 761.5 million tons worldwide (FAOSTAT 2019). Due to its nutritional value, wheat is ranked first for 36% of the world’s population and representing the most important staple food. It provides 20% of calorie supplies and 55% of carbohydrate demands globally, as well as containing pivotal micronutrients (Chattha et al., 2017; Mourad et al., 2019). In many developing countries, wheat contributes more than 50% of the calorific supply daily (Cakmak et al., 2010; Bhavani et al., 2021). It was predicted that wheat will not meet the production demands by 2050 and that climate stresses will further exacerbate this problem (Reynolds et al., 2021). The global productivity of wheat should be increased by nearly 70% to meet the high demand for wheat by 2050. However, wheat production and productivity are threatened by various abiotic stresses. Among other abiotic stresses, salinity is a major problem to agriculture. More than 20% of agricultural land is currently affected by salinity, and it is already affecting almost 954 million hectares of the world’s total land area (Shahid et al., 2018; Bhavani et al., 2021; Hafeez et al., 2021). For developing economies such as Egypt, this critical situation has an enormous impact. The current production demand for wheat in Egypt is not being met. Thus, expanding wheat growth is necessary in newly reclaimed areas due to limited areas of agricultural land. However, these newly reclaimed areas suffer from some abiotic stresses, especially salinity. Wheat is a moderately salt-tolerant crop (Chattha et al., 2017; Dawood et al., 2021; Hafeez et al., 2021) and has wide genotypic differences in salinity tolerance (Saqib et al., 2005). Recent studies state that physiological traits have the potential to improve crop performance under abiotic stress. The physiological basis for salinity tolerance is still poorly understood. Therefore, a better understanding of the genetic basis of physiological trait variability will improve the efficiency of wheat for salinity tolerance. In general, the expression of any physiological trait is influenced by the underlying genetic make-up (G), the surrounding environment (E), and their interactions (G×E).

Destructive evaluation techniques for chlorophyll content (CC) and specific leaf area (SLA) are laborious, time-consuming, and expensive. These techniques became less suitable, as the ultimate goal of the breeding program is to select more resilient genotypes by screening large numbers of genotypes for various desirable traits, including morphophysiological traits. Therefore, non-destructive techniques, such as spectral reflectance, chlorophyll measurement, stomatal conductance, and SLA estimation are more relevant for this task because they are fast, cheap, and reliable. Phenotyping for breeding for physiological traits includes genetically complex physiological traits, such as osmotic adjustment, accumulation and remobilization of stem reserves, superior photosynthesis, heat- and desiccation-tolerant enzymes, canopy temperature, and root system architecture, as well as phenomics and genomic approaches (Reynolds & Langridge, 2016). To improve genetic gains for different desirable traits, spectral-based measurement can be employed as a selection tool in plant breeding (Babar et al., 2006).

To screen for salinity tolerance in wheat, chlorophyll content and leaf elongation are non-destructive and quick parameters (Munns & James, 2003). It is important to select an index for salinity tolerance in wheat (Ma et al., 2006). Chlorophyll content is degradable under salinity stress. Thus, it has been employed as a selection criterion for salt tolerance in cereal crops such as barley and rice (Mahlooji et al., 2018). It can be used to indirectly select high-yielding genotypes under salinity stress in wheat (Kiani-Pouya and Rasouli, 2014). Retaining a high level of chlorophyll contributed to salinity tolerance in rice (Nounjan et al., 2020). The canopy temperature (CT) is cost-effective, quick, non-destructive, and easy to use to estimate the whole-plant response. However, the reasonability of CT varied when tested under different environments (Royo et al., 2002). Under various stressful environments, CT exhibited a strong correlation with yield-related attributes in wheat. Thus, CT can be used as an indirect indicator to select for yield improvement under stressful environments (Pierre et al., 2010). Under drought stress, CT explained 60% of yield variation, indicating that it is a suitable tool to select for yield attributes in the recombinant inbred line (RIL) wheat population (Trethowan and Reynolds, 2007), and it is a potential parameter to select for salinity tolerance in bermudagrasses (Tran et al., 2018). As a non-destructive trait, CT discriminated the salt-tolerant from the salt-sensitive wheat cultivars (Munns & James, 2003). In terms of salinity tolerance, two contrasting wheat cultivars exhibited different CT values under salinity stress, with the sensitive cultivars having a low osmotic adjustment that showed an increase in CT compared with the tolerant cultivars that had a high osmotic adjustment (Sharbatkhari et al., 2016). In contrast to Egyptian wheat cultivars (application of infrared thermal imagery for monitoring salt tolerant of wheat genotypes), CT negatively and significantly correlated with the status of plant water under salinity stress. Under stressful growth conditions such as moisture, CC values measured by Soil Plant Analysis Development (SPAD), along with CT, are suitable screening tools (Jain et al., 2014).

Possessing a high green SLA in the post-anthesis stage maintains carbon assimilation and contributes to grain-filling (Rahimi et al., 2011). SLA has been successfully used to discriminate the salinity tolerance in desert grass versus bermudagrasses (Marcum et al., 2005). Similarly, SLA has been considered to be a reliable selective criterion for salt tolerance in wheat under both field and greenhouse conditions (Elahi et al., 2020). Leaf area was found to be positively correlated with grain-related traits, such as thousand-grain weight and panicle weight in cereals (Yue et al., 2006).

The genome wide association study is considered to be one of the most useful approaches that aims to identify candidate genes for biotic and abiotic stress tolerance (Abou-Zeid & Mourad, 2021; Ahmed et al., 2021; Mourad et al., 2018; Moursi et al., 2020; Thabet et al., 2021, 2021b). It tests the significant association between the genomic regions and marker (such as SNP) with a target trait in the tested populations. Subsequently, these markers are used to identify candidate genes that tailor to the variation of the trait(s) of interest (Alqudah et al., 2020). Several quantitative trait loci were identified under various abiotic stresses in wheat for CC. Seventeen QTLs were mapped on chromosomes 2A, 2B, 2D, 5B, and 7A in a recombinant inbred line (RIL) population under heat stress (Quarrie et al., 2006). Likewise, under salinity stress, several sets of QTLs were mapped in populations with different genetic backgrounds. Two QTLs were detected on chromosomes 3D and 7A in a recombinant inbred line population (Ma and Li, 2018). Similarly, four QTLs were localized for chlorophyll content on chromosomes 2D, 5A, 5B, and 5D in a DH population (Genc et al., 2019). Seven QTLs were identified for CC on chromosomes 2B, 6B, 7B, 5A, and 7D in a RIL population (Ghaedrahmati et al., 2014). Five QTLs on chromosomes 1A, 2B, 3D, 7A, and 7A were recently mapped in a RIL population under salinity stress at the seedling stage (Luo et al., 2021).

For CT, several QTL sets were identified under various growth conditions, especially heat and drought. Five QTLs for CT were mapped on chromosomes 2D, 3B, 3B, 5D, and 7A under heat stress in a DH population (Pinto et al., 2010). The authors of this study found that two QTLs were controlling the yield attributes, as well as CT. In another study, six QTLs were detected for heat and drought and were controlling agronomic and physiological traits, including CT. In three RIL populations, 12 QTLs for canopy activity and yield traits clustered together under non-stressful conditions (Li et al., 2021). Five QTLs were mapped on chromosomes 2A, 5A, and 7D for CT under moisture deficient conditions (Puttamadanayaka et al., 2020). No QTLs have been reported for CT under salinity stress to the best of our knowledge. In durum wheat, the QTLs for CC and CT co-localized at the same genomic regions under drought stress. Moreover, the authors found that the QTLs for CT and yield attributes co-localized at the same genomic regions on chromosomes 1B, 2A, 3B, 4B, 5A, 5B, 6A, 6B, and 7B (Diab et al., 2008).

Many QTLs have been mapped under different abiotic stresses for SLA. Twenty QTLs for leaf area-related traits were mapped in a RIL wheat population under diverse water treatments (Yang and Miao, 2010). A QTL on chromosome A7 for yield was found to attribute a variation in SLA and CC under drought in a biparental DH population. In association with higher flag leaf chlorophyll content and wider leaves, alleles of this QTL contributed a 20% yield increase per spike (Quarrie et al., 2006). However, only a few validated QTLs have been reported for physiological traits for the ease, efficiency, and availability of the tools to use physiological traits as phenotyping indicators (Sukumaran et al., 2015; Edae et al., 2018).

Our knowledge about the genetic control of these physiological traits (CC, CT, and SLA) under salinity, as well as the number of QTLs identified for CC, CT, and SLA in wheat, is rather limited compared to other abiotic stresses such as drought and heat so far. Therefore, the objectives of the current study are to 1) estimate the genotypic variation of these physiological traits under salinity stress, and 2) map the QTLs and candidate genes for the corresponding traits.

## Materials and methods

### Plant material

A set of 153 highly diverse spring wheat (*Triticum aestivum* L.) genotypes that are adapted to Egyptian conditions (Ahmed Sallam, personal communications) were used in this study (Mourad et al., 2020). The diverse collection represented 14 different countries, including Egypt, and was obtained from the USDA-ARS worldwide core collection. The list of material is presented in Supplementary Table S1.

### Phenotypic evaluation

The pots experiment was carried out in 2020/2021 winter season in the Experimental Farm of Faculty of Agriculture, Sohag University, Sohag, Egypt. After sowing the grain, all pots were watered with tap water (having EC of 300 ppm) for 20 days. After that, two irrigation treatments (up to field capacity) were applied. The first group of pots was irrigated with tap water (as a control) and the second was irrigated with 5,000 ppm of saline water. The plants were imposed with salinity stress from day 20 until the termination of the experiment. All treatments were replicated four times and arranged in a completely randomized block design. Each replication consisted of six grains sown in a 12 kg capacity plastic pot (30 cm in diameter × 32 cm in depth) containing a combination of clay and sandy soil (2:1). The plastic pots were maintained in a greenhouse under natural light, and the temperature was 17–30°C during the day and 6–18°C at night. The saline water used was prepared by adding weighted amounts of NaCl salt to potable water to accomplish the required salinity levels. Fertilizers were uniformly mixed in the soil before filling the pots to provide the equivalent of 238 kg ha^−1^ ammonium nitrate (33.5% N), 75 kg ha^−1^ calcium superphosphate (15.5% P_2_O), and 58 kg ha^−1^ potassium sulfate (48% K_2_O). Each pot contained 12 kg of fertilized soil. The analysis of soil was performed according to Page et al. (1985) (Supplementary Table S2). Post-anthesis and per replicate, five plants were chosen randomly, and data were recorded for CC (mg cm^−2^) using a chlorophyll meter (Soil Plant Analysis Development, SPAD-502, Minolta, Osaka, Japan), CT using an infrared thermometer, and SLA (cm^2^ produced g^−1^ leaf dry weight plant^−1^).

### Statistical analysis

The analysis of variance (ANOVA) was computed under both conditions to evaluate the genotype × treatment (G × T) interaction according to the following equation:

*Y*_*ijk*_ = *μ*+ *g*_*i*_ + *r*_*j*_ + *t*_*k*_ + *gt*_*ik*_ + *tgr*_*ijk*_Where Y_ijk_ is the observation of a genotype i in a replication r tested under treatments k (control vs. salinity stress), µ is the general average, and g_i_, t_k_, and r_j_ refer to the eﬀects of genotypes treatment, and replications, respectively. gt_ik_ is genotype × treatment interaction. grt_ijk_ is the genotype replication × treatment (error).

The broad-sense heritability (H^2^) for the measured traits was calculated according to the equation of (Rasmusson and Lambert, 1961) as follows:

$$H^{2}=\frac{\sigma^{2}G}{\left(\sigma^{2}G+\frac{\sigma^{2}GT}{T}\right)+\frac{\sigma^{2}e}{Tr}}$$

where, σ^2^G is the variance of genotypes (accessions), σ^2^G × treatment (T) is the variance component of the interaction between genotypes G × T, σ^2^e is the variance of error, and r is the number of replicates.

The ANOVA,

$$H^{2}$$,

and phenotypic correlations were computed using PLABSTAT A3 (Utz, 1997).

The figures and presentation of the phenotypic data were performed and plotted using http://www.bioinformatics.com.cn, R software, and Microsoft Excel 2016.

### Genetic analysis

#### DNA extraction and genotyping-by-sequencing

The DNA was extracted from two leaves (2-weeks old seedlings) from all genotypes. The extraction was performed using BioSprint 96 DNA Plant Kits (Qiagen, Hombrechtikon, Switzerland). Following this, all samples were sent to Kansas State University for GBS according to Elshire et al. (2011). DNA was initially digested using the *PstI* and *MspI* enzymes. The sequencing of the pooled libraries was generated by the Illumina, Inc. NGS platforms. The SNP calling was performed using TASSEL 5.0 v2 software GBS pipeline (Bradbury et al., 2007). For SNP calling, the Chinese Spring genome v1.0 was used for a reference genome from the International Wheat Genome Sequencing Consortium (IWGSC). The GBS tags were aligned using Burrows-Wheeler Aligner 43. Generated SNPs were filtered for minor allele frequency (MAF) at less than 5% and the missing data was at less than 20%. All heterozygous loci were considered missing data. After filtration, a final set of 103 genotypes and 11,362 SNPs remained and were used for further genetic analysis.

### Genome wide association study

The genome wide association study (GWAS) between markers and the phenotypic data of CC, CT, and SLA under both conditions was carried out using TASSEL version 5.0 in the current study (Bradbury et al., 2007). The analysis of population structure was extensively studied by Mourad et al. (2020). The general linear model (GLM) + principal component analysis (PCA) model were used in GWAS. A Bonferroni correction with a suggestive *p*-value of 1% (1/total number of markers) was used to test the statistical significance of marker-trait associations (Duggal et al., 2008). Phenotypic effects at the marker loci were calculated as differences between the means of the marker classes. The positive values indicate that the specified marker allele increases the trait, while a negative value indicates that this allele is associated with decreasing the trait. The phenotypic variance explained (R^2^) by significant makers was determined using TASSEL 3.0. All significant QTLs and their position on the chromosomes were illustrated using PhenoGram (http://visualization.ritchielab.org/).

The linkage disequilibrium (*r* ^*2*^) among significant markers located on the same chromosome was calculated using TASSEL v 5.0. The gene annotation for the significant markers was performed to detect the candidate genes using *EnsemblPlants* (https://plants.ensembl.org/Triticum_aestivum/Info/Index). If the significant SNP was located within the candidate gene, it was selected to examine its gene expression. The gene expression at the heading date under abiotic stresses including salt stressed wheat was compared based on the wheat expression database (http://bar.utoronto.ca/).

## Results

### Phenotypic variation

**FIGURE 1:**
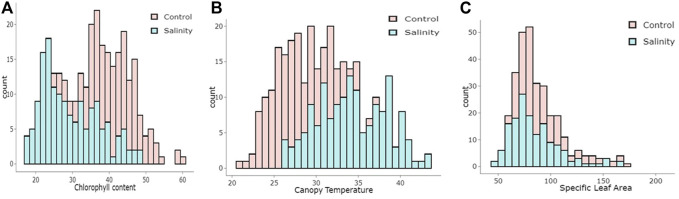
Histogram illustrating the distribution of **(A)** chlorophyll content (CC, mg cm-2), **(B)** canopy temperature **(C)** and specific leaf area (cm2)in wheat under control and salinity stress.

Salinity stress affected the estimated traits differently, as the population’s mean values for CT increased, while they decreased for CC and SLA (Table 1). The mean values of CC were 40.87 mg cm^−2^ under the control and 29.51 mg cm^−2^ under salinity. For CT, the mean values were 27.87°C and 34.23°C under the control and salinity, respectively. For SLA, the mean values were 90.49 cm^2^ g^−1^ for the control and 85.54 cm^2^ g^−1^ for salinity (Tale 1). The minimum and maximum values for all traits under control and salinity are listed in Table 1. The traits CC and CT showed normal distribution, while SLA was relatively skewed (Figure 1).

**TABLE 1:** Ranges, means, standard deviation (SD), coefficient of variation (CV) and analysis of variance (ANOVA) for all traits scored on wheat under control and salinity. Min stands for minimum, Max for Maximum and STI for Salt Tolerance Index.

Trait	Control					Salinity				
	Min	Max	Mean	SD	CV	Min	Max	Mean	SD	CV
Chlorophyll content (CC, mg cm-2)	24.65	59.48	40.87	6.916	16.76	16.95	48.73	29.51	7.791	26.40
Canopy Temperature (CT, ᵒC)	21.18	37.45	27.87	3.295	11.82	26.20	46.46	34.23	4.117	12.03
Specific Leaf Area (SLA, cm2)	60.22	173.50	90.49	23.059	25.48	43.60	166.89	85.54	24.610	28.93
**Analysis of variance (ANOVA)**										
**Source of Variance**	**Treatments**	**Genotypes**	**Replications**	**Treatment × Genotype**	**Heritability**					
Chlorophyll content (CC)	991.16**	73.51**	2.41+	7.52**	98.64					
Canopy Temperature (CT)	589.26**	19.89**	0.82	4.60**	94.97					
Specific Leaf Area (SLA)	3.66+	5.56**	2.06	3.86**	82.02					

^+^, *,**, *** stand for significance levels *p* ≤ 0.1, 0.05, 0.01 and 0.001, respectively.

### Analysis of variance, heritabilities, and correlation

A wide variation has been observed for all traits. The genotypic variation of all traits was always higher than the variation attributed to the genotype-environment interaction. However, high significant genotype treatment interaction was observed for all traits (Table 1). The effect of the treatment was very significant for CC and CT compared to SLA. CC showed the highest variation attributed for all variation parameters, including treatment effect, genotype effect, and genotype environment interaction. The broad-sense heritability estimates were extremely high for all traits, with values of 98.64, 94.97, and 82.02 for CC, CT, and SLA, respectively (Table 1).

**FIGURE 2:**
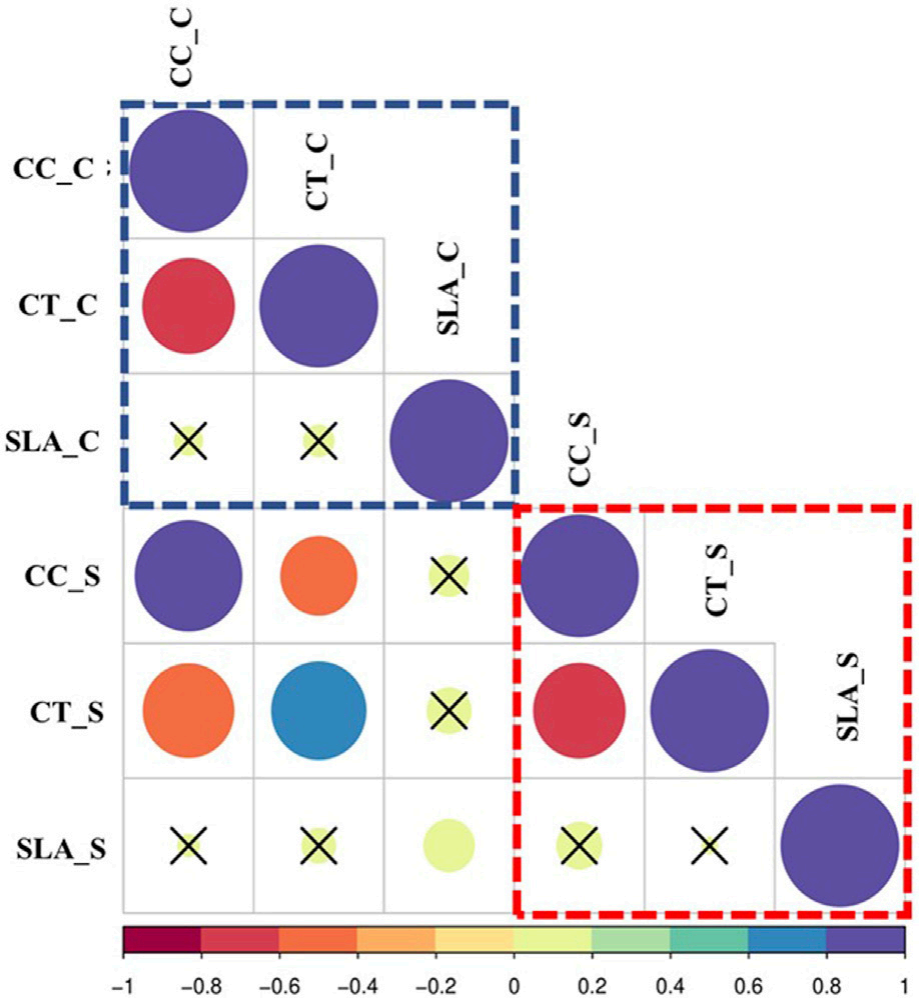
Correlations of the estimated traits, Chlorophyll content (CC), Canopy Temperature (CT), Specific Leaf Area (SLA) in wheat under control and salinity. Red square = Correlations under Salinity stress, Blue square = correlations under control, Green square = Correlations under Salinity. C refers to control, while S refers to salinity.

Correlation coefficients for all traits are illustrated in Figure 2. No positive significant correlations were observed among the traits under the control or salinity. Meanwhile, negative significant correlations were observed under the control between chlorophyll content under control (CC_C) and canopy temperature under control (CT_C) (CT_C) (*r* = -0.65**), as well as between chlorophyll content under salinity (CC_S) and canopy temperature under salinity (CT_S) (*r* = -0.63**). Across both the control and salinity treatments, the elements of each trait showed significant positive correlations for chlorophyll content under control (CC_C) and chlorophyll content under salinity (CC_S) (*r* = 82**), CT_C and CT_S (0.64**), and specific leaf area under control (SLA_C) (SLA_C) and specific leaf area under salinity (SLA_S) (0.18*) (Figure 2).

### Genome-wide association study

**FIGURE 3:**
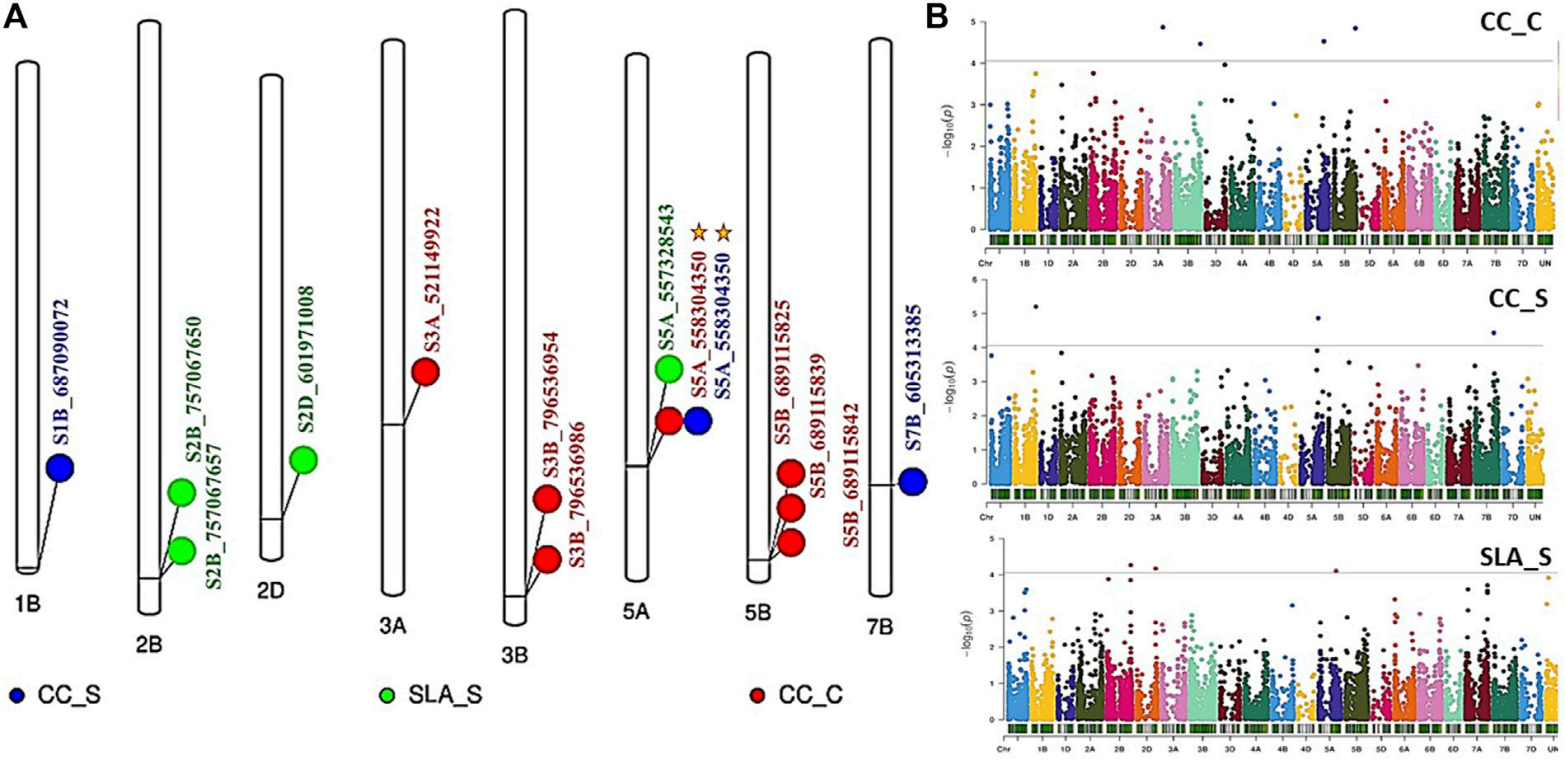
**(A)** The distribution of the significant SNPs detected by GWAS on wheat chromosomes, **(B)** Manhattan plot for all *p*-values in the three traits. Gold star refers to a marker with pleiotropic effects. CC and SLA refer to chlorophyll content under C (control) and S (salinity).

**FIGURE 4:**
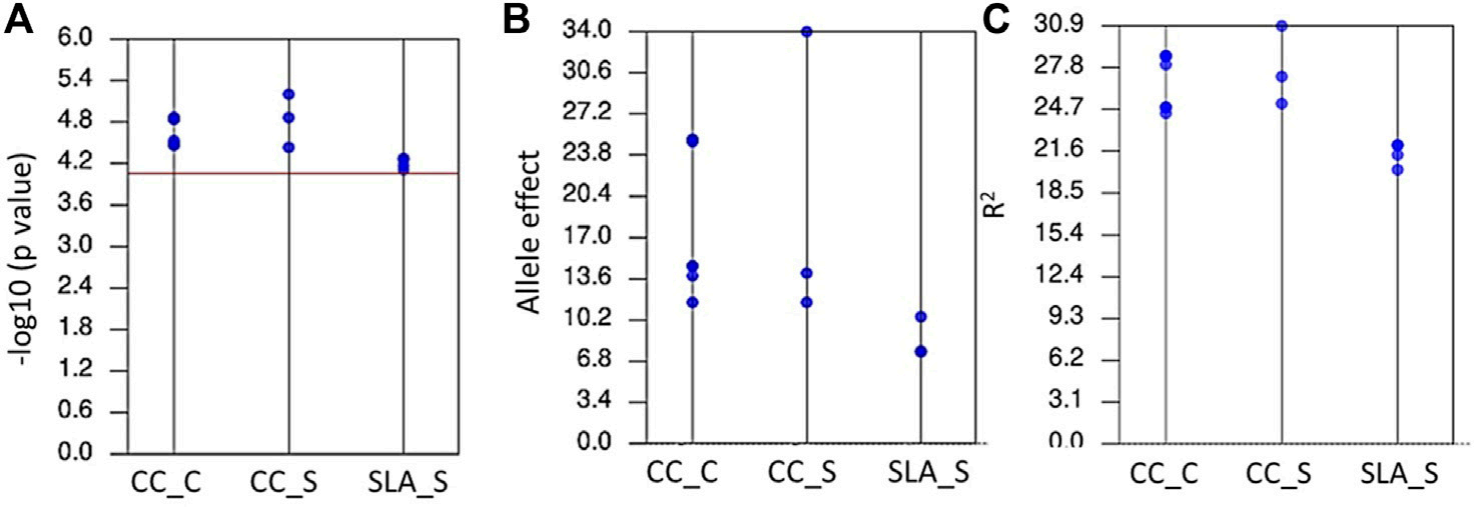
The range of *p*-values **(A)**, allele effects **(B)**, and phenotypic variation explained by markers (R2) **(C)** for all significant markers detected by GWAS for chlorophyll content under control (CC_C), chlorophyll content under salinity (CC_S), and specific leaf area under salinity (SLA_S).

Genome wide association mapping was performed between the 11,362 SNPs and all traits under control and salt stress conditions. The quantile-quantile plots for all traits under the control and salinity using GLM + PCA are presented in Supplementary Figures S1A and S1C. The GWAS analysis revealed 14 significant SNPs distributed on 1B, 2B, 2D, 3A, 3B, 5A, 5B, and 7B (Figure 3A). One significant SNP was located on 1B (CC_S), 2D (SLA_S), 3A (CC_C), and 7B (CC_S). Chromosomes 2 and 3B had two SNPs associated with SLA_S and CC_C, respectively. Three SNPs were located on chromosomes 5A and 5B. Seven SNPs were found to be highly associated with CC under control conditions, while three and four significant SNPs were associated with CC_S and SLA_S under salt stress, respectively. The Manhattan plot for marker-trait association is illustrated in Figure 3B for each trait. No significant SNPs were found to be associated with CT under both conditions and SLA_C. Detailed results of the GWAS are presented in Table 2. The *p*-values ranged from 6.27E-06 (S1B_687090072, CC_S) to 7.77E-05 (S5A_557328543, SLA_S) (Table 2; Figure 4A). The allele effects of the target allele associated with increased traits are presented in Figure 4B. The phenotypic variation explained by each marker (R^2^) ranged from 24.88% to 28.67%, 25.15%–30.90%, and 20.26%–22.08% for CC_C, CC_S, and SLA_S, respectively (Figure 4C).

**TABLE 2:** Genome-wide association analysis for chlorophyll content (CC) and specific leaf area (SLA) scored under control (C) and salinity (S) conditions.

Trait	Marker^a^	Chr	Pos	*p-value*	R^2^ (%)^b^	SNP1^c^	AE^d^	Gene	Coded protein
CC_C	S3A_521149922	3A	521149922	1.35E-05	28.03	C/G	13.84	—	—
	S5B_689115825	5B	689115825	1.43E-05	28.67	**A/T**	7.60	—	—
	S5B_689115839	5B	689115839	1.43E-05	28.67	**A/G**	7.60	—	—
	S5B_689115842	5B	689115842	1.43E-05	28.67	**G/T**	7.60		
	***S5A_558304350***	5A	558304350	2.95E-05	24.44	**A/T**	14.06	TraesCS5A02G355900	P-loop containing nucleoside triphosphate hydrolase
	S3B_796536954	3B	796536954	3.41E-05	24.88	**C/T**	14.64	—	—
	S3B_796536986	3B	796536986	3.41E-05	24.88	**C/T**	14.64	—	—
CC_S	S1B_687090072	1B	687090072	6.27E-06	30.90	**A/C**	13.84	TraesCS1B02G479100	Guanine nucleotide binding protein (G-protein), alpha subunit
	***S5A_558304350***	5A	558304350	1.37E-05	27.16	**A/T**	15.09	TraesCS5A02G355900	P-loop containing nucleoside triphosphate hydrolase
	S7B_605313385	7B	605313385	3.69E-05	25.15	**A/G**	10.46	—	—
SLA_S	S2B_757067650	2B	757067650	5.40E-05	22.08	**A/G**	7.605	—	—
	S2B_757067657	2B	757067657	5.40E-05	22.08	**C/T**	7.605	—	—
	S2D_601971008	2D	601971008	6.70E-05	20.26	**A/G**	7.605	TraesCS2D02G509500	Wall-associated receptor kinase, galacturonan-binding domain
	S5A_557328543	5A	557328543	7.77E-05	21.36	**C/T**	10.46	—	—

a Bold-italic marker refers to markers with pleiotropic effects.

b Phenotypic variation explained by markers.

c Red allele refers to the target allele which associated with increased the trait.

d The effect of target allele (red allele).

**FIGURE 5:**
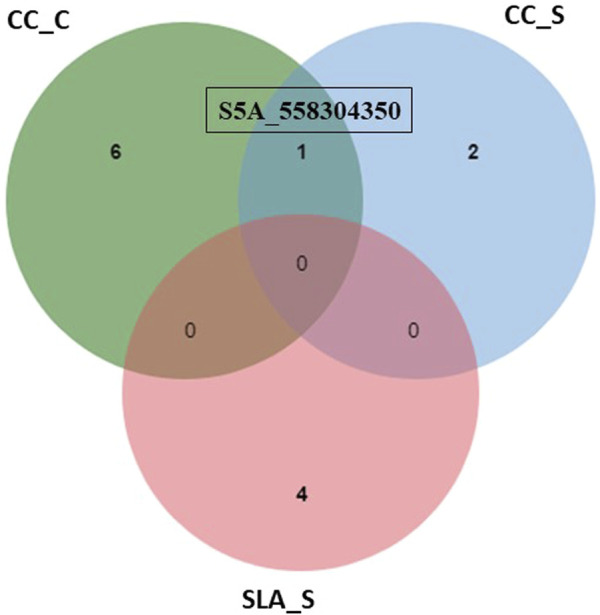
Number of individual and common significant SNPs detected by GWAS for chlorophyll content under control (CC_C), chlorophyll content under salinity (CC_S), and specific leaf area under salinity (SLA_S).

Among all SNPs, one significant SNP associated with CC_S and CC_C (Figure 5). This SNP was located on chromosome 5A. Allele A was associated with increased CC and SLA under both conditions. The effect of this allele was higher under salinity (15.09%) conditions compared to the control (14.16%). Moreover, it had a higher R^2^ value under salinity (27.16%) conditions compared to the control (24.44%).

**FIGURE 6:**
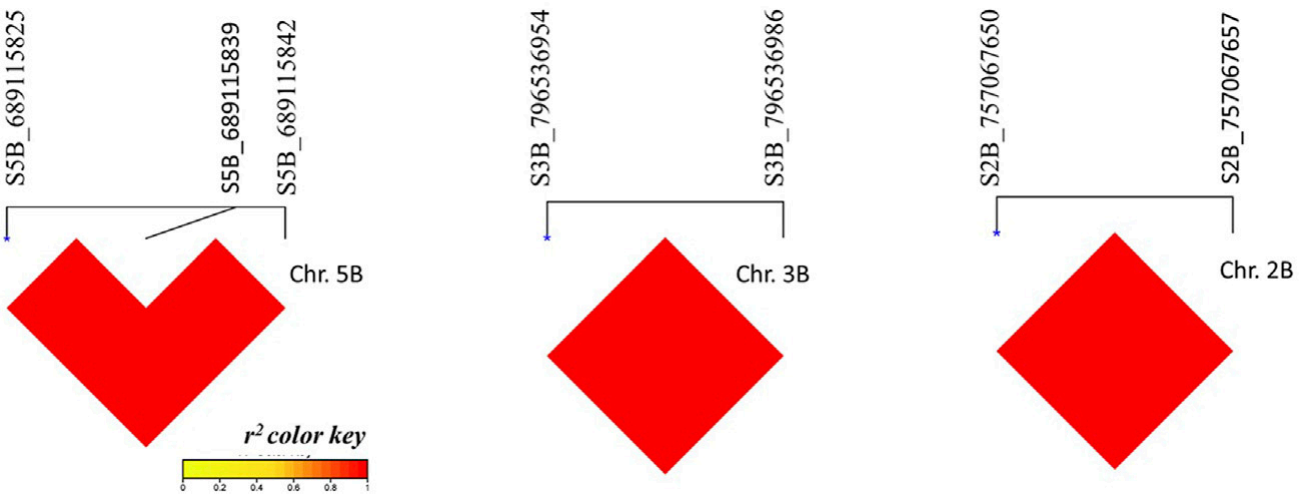
I,inkage disequilibrium r2 among significant SNPs located on the same chromosome. Red refers to complete 1.1)

The linkage disequilibrium was calculated among SNPs located on the same chromosome. A high and complete LD (*r* ^*2*^ = 1) was found among all SNPs located on chromosomes 5B, 3B, and 2B (Figure 6).

**FIGURE 7:**
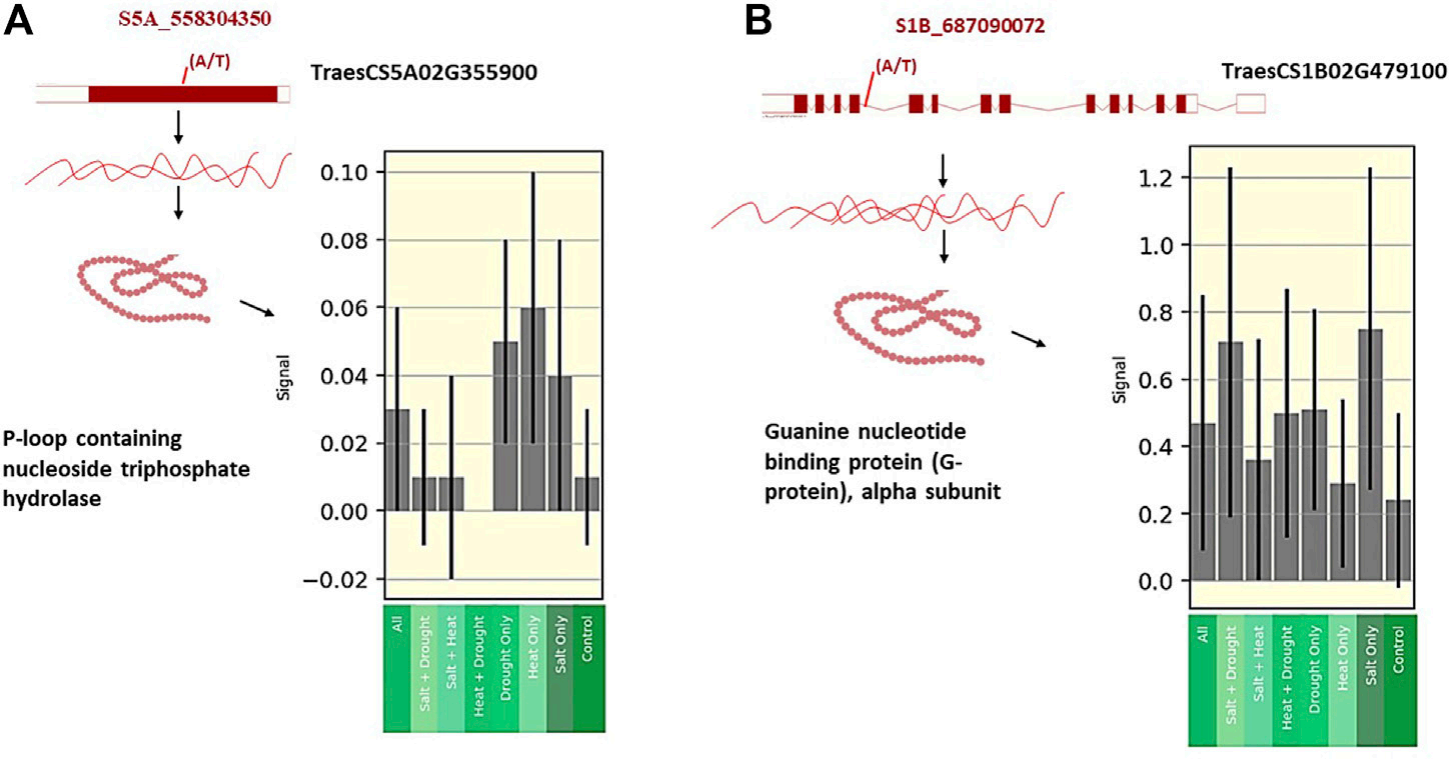
**(A)** Gene expression of TaesCs5AG355900 at flag leaf under different abiotic stress after heading date. **(B)** Gene expression of InesCS1B026479100 at flag leaf under different abiotic stress after heading date.

To confirm the association between markers and traits, all SNPs were annotated in the wheat genome. Three out of 14 SNPs fell into three gene models (Table 2). The expression levels of these three genes were obtained from the wheat expression database (http://bar.utoronto.ca/). The SNP marker (S5A_558304350 SNP) associated with chlorophyll content under control (CC_C) and chlorophyll content under salinity (CC_S) was found to fall within the *TraesCS5A02G355900* gene model. This gene encodes P-loop containing nucleoside triphosphate hydrolase that was found to have a high expression in the flag leaf after the heading date under salt stress compared to the control (Figure 7A). Moreover, the SNP marker (S1B_687090072) associated with CC_S was located within the *TraesCS1B02G479100* gene model which encodes the Guanine nucleotide-binding protein (G-protein) alpha subunit. After the heading date, this protein showed a remarkably high expression under salt stress compared to the control with more than three folds (Figure 7B) in the flag leaf stage. The S2D_601971008 SNP controlling SLA_S was found to fall within TraesCS2D02G509500, which encodes the Wall-associated receptor kinase (WAK), galacturonan-binding domain. No expression data was found for this protein under either the control or salt stress.

It is noteworthy that the SNP marker (S5A_558304350) showed a plausible constitutive expression pattern because it exhibited an association with CC_C and CC_S (Table 2; Figure 3A, Figure 3).

## Discussion

### Genetic variation in chlorophyll content, canopy temperature, and specific leaf area under normal and salinity conditions

Improving salt tolerance is a great challenge as it is a very complex trait that is under polygenic control. More study is required on more morphological and physiological traits to understand the complexity of salt tolerance (Mourad et al., 2019; Moursi et al., 2020; Mondal et al., 2021; Thabet et al., 2021). The high genetic variation existing among genotypes in the three traits was extremely useful for plant breeders in selecting genotypes with increased CC_S and decreased CT_S in the current study. The high significant G ×T interaction indicated the different responses of the genotypes to different saline conditions. The high significant differences between the control and salinity conditions indicated that the genetic variation was maintained in the current collection. The high genetic variation and heritability of the studied traits indicated that CC, CT, and SLA could all be selected under salinity conditions. Such high heritability for the traits were due to the accurate measurements conducted by digital equipment. In addition, the experiment was semi-controlled with very slight changes in temperature and humidity which did not affect the repeatability of data across the three replication in the two treatments.

The CT increased, whereas the CC decreased in the plants treated with salinity compared to the control (Table 1). This finding agrees with what was found in soybean plants concerning the increase in CT and decrease in CC due to salinity stress (Chung et al., 2020). Excessive salinity causes a reduction in the CC of leaves and, subsequently, the net photosynthesis (Sheng et al., 2008; Chung et al., 2020).

The significant negative correlations between CC and CT under both the control and salinity conditions (Figure 2) are explained by the ability of the plants to keep the stomata open to sustain photosynthesis, resulting in the CT being lowered. Salinity stress decreased the net photosynthesis and transpiration but increased the CT. The reduction in CC caused a reduction in photosynthesis. CT is a good indicator for plant status under abiotic stresses, as it indicates the variation of photosynthesis and transpiration rates. Similar results were reported in soybean plants (Chung et al., 2020). The salt-tolerant wheat genotypes revealed high levels of CC compared to the salt-sensitive group. Thus, chlorophyll content would be useful in screening large numbers of genotypes (Mansour et al., 2020). Genotypes showing a ‘cooler’ CT had a better water status and were found to be associated with yield traits (Mahlooji, 2021).

However, salinity stress reduced CC more than SLA, and they are negatively correlated, which might be due to the disintegration of chlorophyll pigment under salinity. The leaf area was slightly affected. In peanut plants under drought stress, SLA correlated negatively and significantly with chlorophyll content (Wang et al., 2004; Mansour et al., 2020). The authors concluded that SLA and CC are reasonable selection criteria for drought stress.

Several studies reported that both CC and CT contribute positively to the final yield. The grain yield per plant had a significant positive correlation with CT, as well as CC (Elahi et al., 2020). The authors suggest that these traits can be used as selection tools for yield improvement under drought. The flag leaf width was found to be associated with grain yield per ear, as well as the number of grains per ear (Reynolds et al., 2005; Quarrie et al., 2006), suggesting a positive role for leaf-related attributes in the final grain yield. High net photosynthesis rates per unit SLA were observed without any alteration in the distribution of CC or SLA during grain filling. This could explain the negative and significant correlations between SLA and CC across both treatments (Reynolds et al., 2005). The SLA_S was reduced in the current study, and our findings agree with the results that were reported in other plant species, such as the melon, hop pepper, spinach, purslane, and wheat (Rahimi et al., 2011; Sarabi et al., 2017; Ziaf et al., 2021). This reduction in SLA can be explained as an adaptation to salinity stress by reducing the transpiration rate and water loss. Low SLA is associated with salt tolerance via the accumulation of high levels of dry matters, as well as secondary metabolites per unit of leaf area, enabling plants to cope with prolonged stressful conditions (Wang et al., 2016; Tabassum et al., 2017).

### Genome wide association study

The GWAS used 11,362 SNP markers covering the wheat genome to identify candidate genes associated with CC, CT, and SLA in this study. The genotyping-by-sequencing method generates thousands of SNPs that can be used to genetically dissect the complexity of target traits such as salt tolerance, which is one of its advantages (Hussain et al., 2017; Mourad et al., 2018; Eltaher et al., 2021).

The analysis of population structure for the current population was extensively described by Mourad et al. (2020), revealed three subpopulations. Therefore, GLM + PCA was used in this study to avoid spurious association resulting from the structure. The mixed linear model (MLM) + kinship was also used to test the association. However, the QQ plot revealed that this model overcorrected the population structure (Supplementary Figures S1B and S1D). As a result, GLM + PCA was the appropriate model for the GWAS as the observed and expected *p* values of all markers are on or near the middle line between the x-axis and the y-axis except the significant markers. All significant markers were detected using Bonferroni correction, which controls false positive association.

The GWAS in this study revealed 14 marker trait associations for CC_C, CC_S, and SLA_S. All SNPs had *R* ^2^ > 20%, indicating they had a major effect on the CC under both treatments and SLA_S. QTL with an *R* ^2^ > 10% can be considered as having major effects on the target traits (Gouy et al., 2014; Maccaferri et al., 2015; Sallam et al., 2016; Hussain et al., 2017; Liu et al., 2019; Mourad et al., 2020). All significant SNPs located on the same chromosome were in a complete LD indicating that these SNPs tend to be co-inherited together and represent the same genomic region, whereas the other significant SNPs represent an individual QTL.

CC at the anthesis stage is an important physiological trait under abiotic and biotic stress conditions, as it plays a critical role in the efficiency of plant photosynthesis (Mourad et al., 2018; Dawood et al., 2020). The GWAS revealed seven QTLs controlling CC under the normal condition on chromosomes 3A, 5B, 5A, and 3B in this study. The SNPs located on chromosomes 5B (three SNPs) and 3B (two SNPs) were in high LD. On the other hand, three QTLs were found under salt stress conditions at the anthesis stage and were located on chromosomes 1B, 5A, and 7B. Many QTLs were reported for CC at the early and vegetative stages, but very few were reported at the anthesis stage under salinity stress. Chaurasia et al. (2021) reported one QTL (AX-94820097) with minor effects (*R* ^2^ = 3.16%–7.82%) for the salt tolerance index of CC at the anthesis stage. This QTL was located on chromosome 6D. A recent study of Alotaibi et al. (2021) found 10 QTLs associated with CC under both low and high salinity conditions at the anthesis stage on chromosomes 7A, 2B, 4A, 5B, 7B, and 5A. The R^2^ of these QTLs ranged from 5.12% to 12.19% under low salinity conditions and from 7.11% to 14.53% under high salinity conditions. Interestingly, we reported a significant SNP (S5A_558304350) located on chromosome 5A (558304350 bp) in this study. This SNP marker (S5A_558304350 SNP) showed a constitutive gene expression as it exhibited an association with CC under both control and salinity stress (Table 2; Figure 3A, Figure 3). This indicates that this SNP is highly valuable due to its ability to be targeted for selection even under control conditions.

The QTL (BS00075959_51) revealed by Alotaibi et al. (2021) on chromosome 5A was located at 588741059, near the SNP found in this study. The significant SNP marker (S5A_558304350) was found to be associated with CC under both conditions, indicating that there was a strong association under different conditions. Allele A of this SNP was associated with increased CC_C (14.06%) and CC_S (15.09%). On average, this allele increased CC_S by 6.8%. This SNP was associated with gene model *TraesCS5A02G355900*. This gene was found to be highly expressed under salt stress in Arabidopsis plants (Kocourková et al., 2011; Chen et al., 2015). Various abiotic stresses, including salt stress, increased the transcription level of phosphatase (Abscisic Acid) ABA *Insensitive*-2 and a transcriptional activator CBF1(*At4g25490*) exclusively in the leaves of Arabidopsis plants (Kreps et al., 2002). The *TraesCS5A02G355900* encodes P-loop containing nucleoside triphosphate hydrolase, which is involved in the response to salt stress (Zhao et al., 2007). Remarkably, this gene had a higher level of expression under salt stress than control conditions in the flag leaf at the heading stage in wheat. Likewise, it had a higher expression under heat and drought compared to salt stress, indicating the critical role it plays in alleviating the effect of various abiotic stresses on wheat leaves (Kocourková et al., 2011; Chen et al., 2015). Another important SNP marker (S1B_687090072), located in the *TraesCS1B02G479100* gene model (*GPA*1 gene), encodes the G-protein alpha subunit. The G-protein enhances salt tolerance in Arabidopsis plants (Lu et al., 2018). Interestingly, this gene was found to have a remarkably high expression under salt stress compared to other abiotic stresses and the control in flag leaves at the heading stage. The SNP marker (S1B_687090072) was located on chromosome 1B at 687090072 pb. In Oyiga et al. (2018) study, a wsnp_Ex_c955_1827719 SNP marker was found to be associated with leaf chlorophyll fluorescence with *R* ^2^ = 4.8% and was located on chromosome 1B at 688768186 pb near the SNP (S1B_687090072) detected in our study. Five significant SNPs associated with salt tolerance index for leaf CC at the vegetative stage were located on chromosomes 2A, 2B, 3B, 4A, and 7A (Chaurasia et al., 2020).

Four significant SNPs were found to be associated with SLA_S. No earlier studies reported GWAS results for this trait. Therefore, these four QTLs can be considered as new and novel in controlling this important trait under salinity in spring wheat. The two SNPs located on chromosome 2B had a complete linkage and represented the same genomic regions/QTLs. All QTLs detected for SLA_S had minor effects. Out of the four SNPs, one SNP (S2D_601971008) was found to be within the *TraesCS2D02G509500* gene model, which encodes the WAK galacturonan-binding domain. The role of WAK is still experimentally unknown, although it has been shown to contribute to the salt stress response (Liu et al., 2021). This was confirmed by lacking gene expression experiments under salt and control conditions (Wheat eFP Browser (utoronto.ca)). It was recently reported that salinity stress-induced de-methyl-esterification of pectin activates stress signaling pathways, which may provide direction in studying the roles of WAKs in the salt stress response (Gigli-Bisceglia et al., 2020). Therefore, reporting the association of this gene with SLA_S could provide a valuable and novel piece of information on the role of WAKs in enhancing salt tolerance in wheat leaves.

The SNP markers detected in this study specifically (S5A_558304350, S1B_687090072, and S2D_601971008) can be converted to Kompetitive allele-specific PCR (KASP) markers for further genetic validation and association with salt tolerance under different genetic backgrounds.

The results of GWAS provided SNP markers for important physiological traits associated with wheat yield under salt stress. The results of this study can be used to improve salt tolerance in wheat and further genetic studies such as genomic selection which can be predict performance of other genotypes using significant markers resulted from genome-wide association study (Sandhu et al., 2021)

## Conclusion

Understanding the genetic control of physiological traits under salinity conditions is a particularly important task for improving salt tolerance in wheat at the anthesis stage, which is important for grain filling duration. Important SNPs controlling CC_S were reported and found to be very similar to previously published genomic regions associated with leaf CC in wheat at the anthesis stage. Novel significant SNPs were detected in SLA_S for the first time in this study. The results of gene annotation and expression highlighted two important SNPs, S5A_558304350 and S1B_687090072, which fell within two gene models that had an extremely high expression under salinity conditions. These results shed light on the power of GWAS analysis used to detect important genes controlling some physiological traits under salinity conditions in this study. The markers detected should be converted to KASP markers for further validation before using them in marker-assisted selection (MAS).

Declarations.

## Data Availability

The original contributions presented in the study are included in the article/Supplementary Material, further inquiries can be directed to the corresponding author.
