# Supplementary material for: Association mapping and candidate genes for physiological non-destructive traits: Chlorophyll content, canopy temperature, and specific leaf area under normal and saline conditions in wheat
Source: Front Genet. 2022 Sep 30;13:980319. doi: 10.3389/fgene.2022.980319 (PMC9561097; doi:10.3389/fgene.2022.980319)
Supplement: Supplementary file 1 [file DataSheet1.ZIP › supp files/supp figures.pptx]

## Slide 1
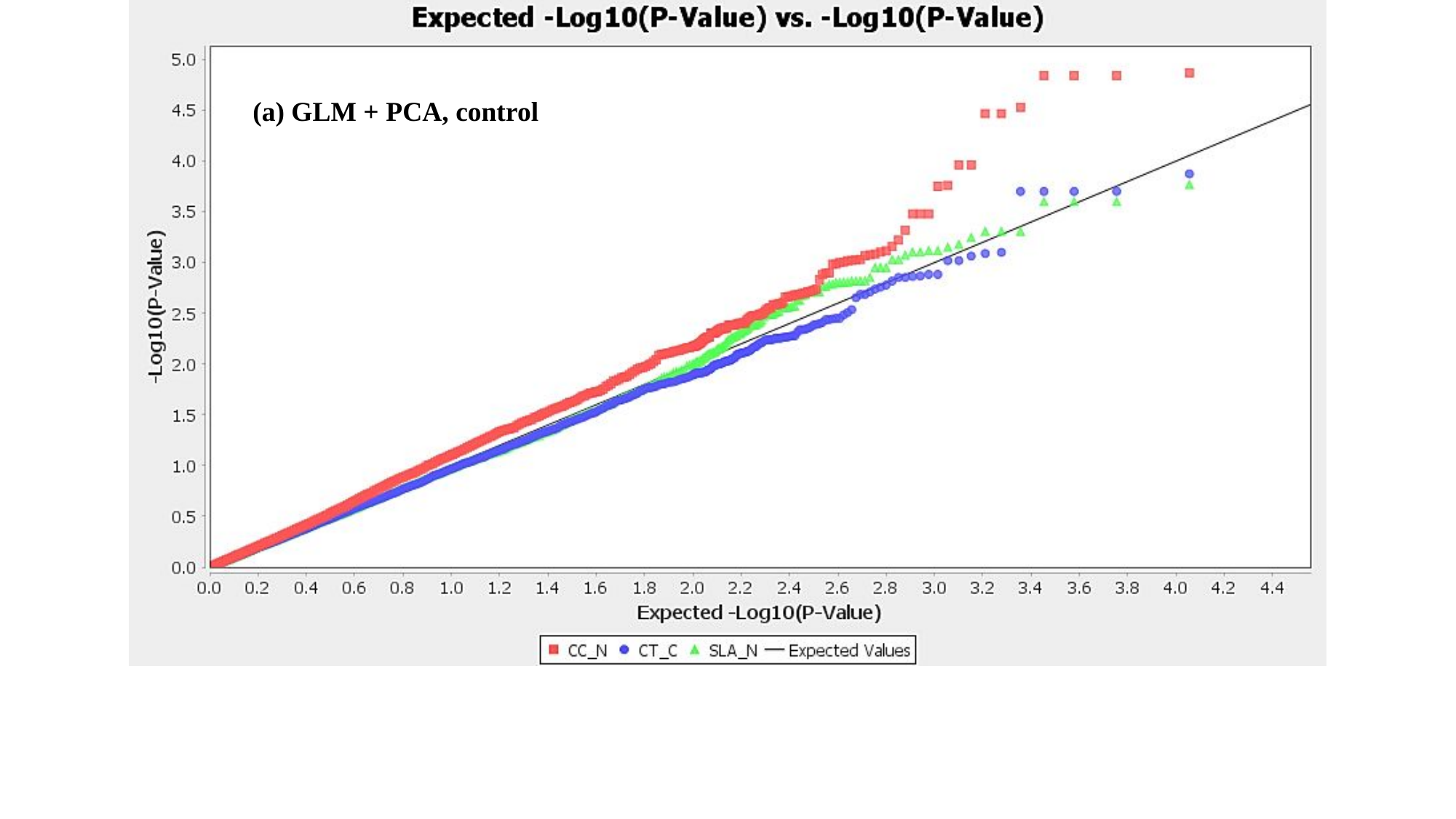

(a) GLM + PCA, control

## Slide 2
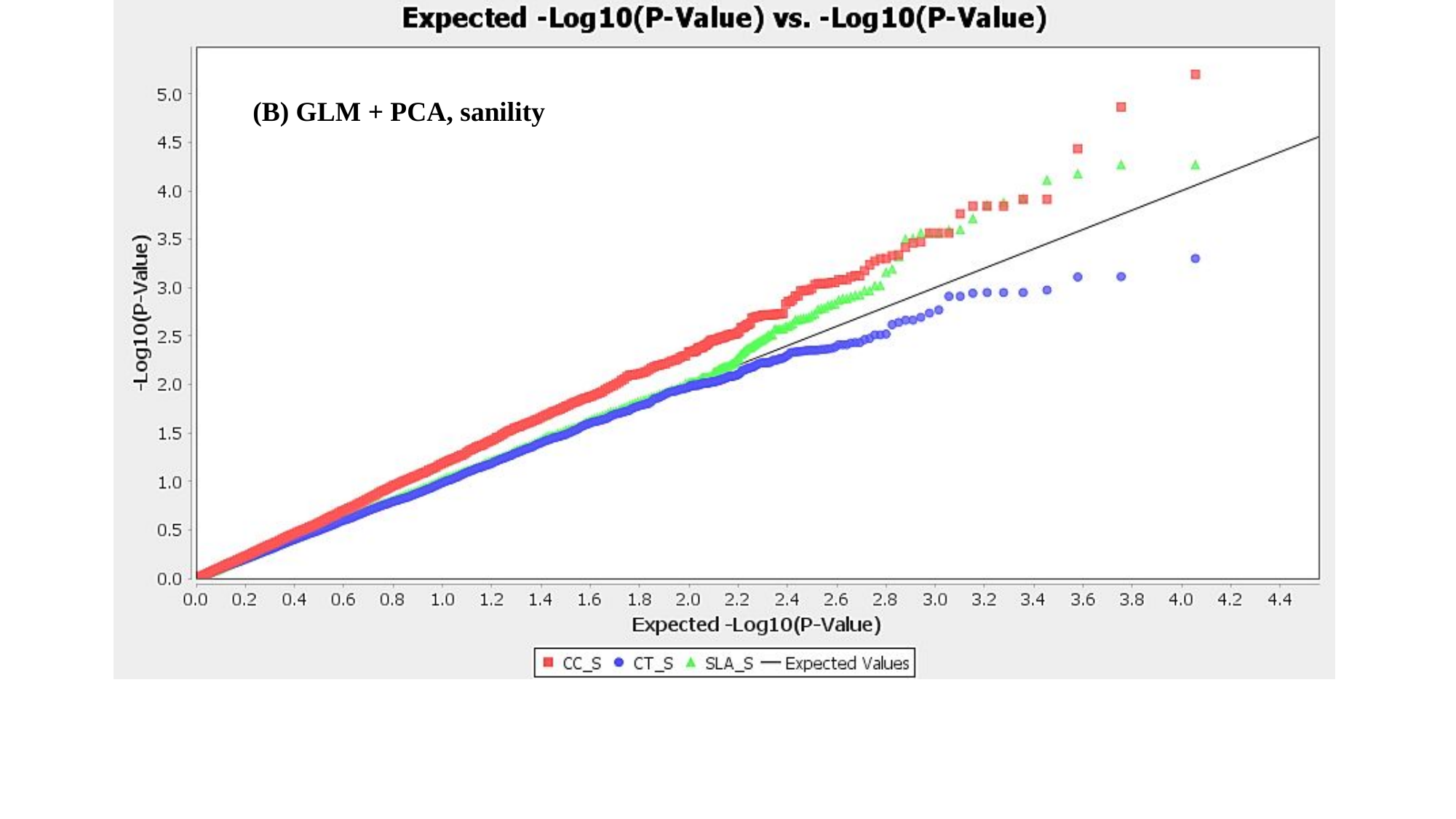

(B) GLM + PCA, sanility

## Slide 3
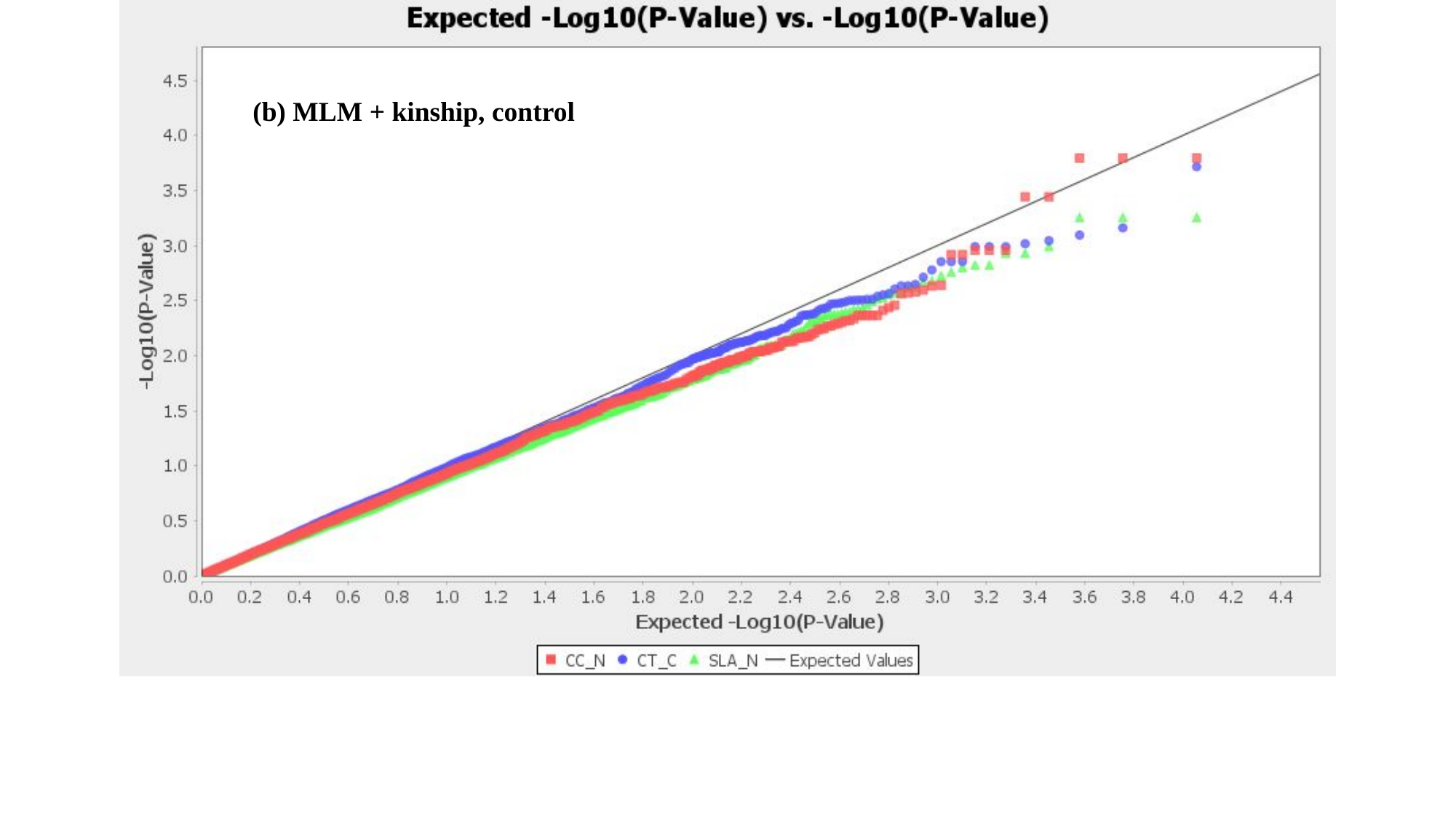

(b) MLM + kinship, control

## Slide 4
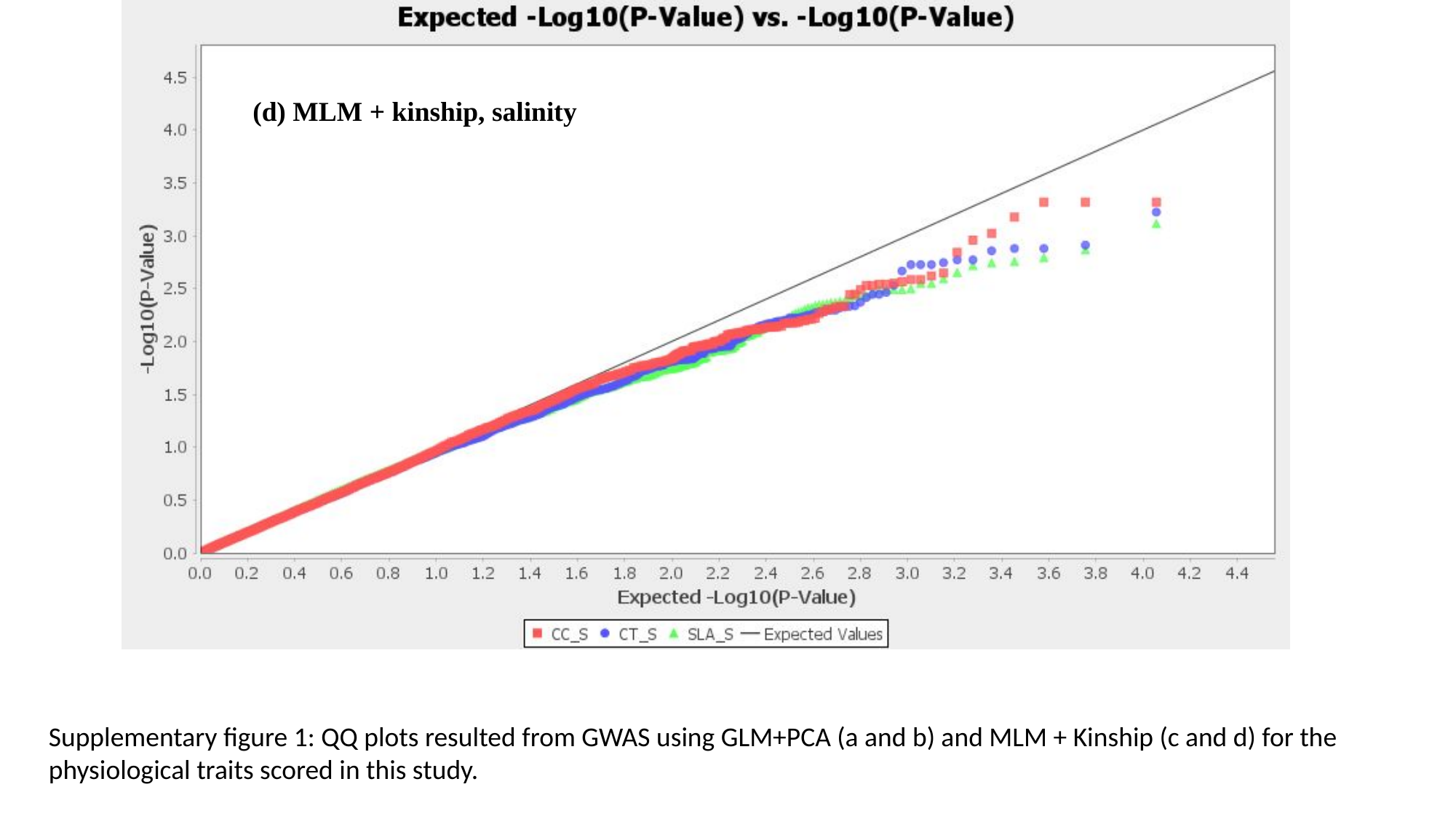

(d) MLM + kinship, salinity
Supplementary figure 1: QQ plots resulted from GWAS using GLM+PCA (a and b) and MLM + Kinship (c and d) for the physiological traits scored in this study.
