# Supplementary material for: Association mapping and candidate genes for physiological non-destructive traits: Chlorophyll content, canopy temperature, and specific leaf area under normal and saline conditions in wheat
Source: Front Genet. 2022 Sep 30;13:980319. doi: 10.3389/fgene.2022.980319 (PMC9561097; doi:10.3389/fgene.2022.980319)
Supplement: Supplementary file 1 [file DataSheet1.ZIP › supp files/Table 1.DOCX]

Supplementary Table 2. Chemical characteristics of the soil before and after cropping.

| Property | Unit | before cropping | After cropping |
| --- | --- | --- | --- |
| pHe | ……. | 7.60 | 8.22 |
| EC_e_ | dSm^-1^ | 1.88 | 5.67 |
| Ca^2+^, | mgl^-1^ | 204 | 607 |
| Mg ^2+^, | mgl^-1^ | 14.58 | 42.67 |
| Na^+^ | mgl^-1^ | 139.13 | 414.78 |
| K^+^ | mgl^-1^ | 29.50 | 85.52 |
| HCO_3_^-^ | mgl^-1^ | 163.92 | 485.42 |
| Cl^‑^ | mgl^-1^ | 320.82 | 970.56 |
| SO_4_^-2^ | mgl | 336.32 | 1003.28 |
| CaCO_3_, % | % | 0.67 | 0.54 |
| Organic matter | % | 0.40 | 0.43 |
| Field Capacity | % | 17 | 18 |
| Wilting Point | % | 10 | 10 |
| Water holding capacity | % | 9 | 10 |
| Sand | % | 35.5 | 39.6 |
| Silt | % | 27.7 | 24.7 |
| Clay | % | 37.9 | 35.6 |
| Texture class | | Clay loam | Clay loam |
